# Supplementary material for: Nivel Corona Cohort: A description of the cohort and methodology used for combining general practice electronic records with patient reported outcomes to study impact of a COVID-19 infection
Source: PLoS One. 2023 Aug 22;18(8):e0288715. doi: 10.1371/journal.pone.0288715 (PMC10443834; doi:10.1371/journal.pone.0288715)
Supplement: S1 Fig — (DOCX) [file pone.0288715.s001.docx]

**S1 Figure. Prevalence of symptom burden marked to severe**
