# Supplementary material for: Nivel Corona Cohort: A description of the cohort and methodology used for combining general practice electronic records with patient reported outcomes to study impact of a COVID-19 infection
Source: PLoS One. 2023 Aug 22;18(8):e0288715. doi: 10.1371/journal.pone.0288715 (PMC10443834; doi:10.1371/journal.pone.0288715)
Supplement: S1 Table — (DOCX) [file pone.0288715.s002.docx]

**S1 Tables. Description of questionnaire and electronic health record data**

| S1A Table. Description of questionnaires | | | | | | |
| --- | --- | --- | --- | --- | --- | --- |
| Topic |  | **Description** | **Q1** | **Q2** | **Q3** | **Q4** |
| Patient  characteristics |  | Age | x | x | x | x |
|  | - | Sex | x | x | x | x |
|  | - | Highest completed level of education | x |  |  |  |
|  | - | Marital status | x |  |  |  |
|  | - | Living arrangement | x |  |  |  |
|  | - | Country of birth | x |  |  |  |
|  | - | Pregnancy |  |  | x |  |
| Resilience | - | Resilience Evaluation Scale (RES): self-reported psychological resilience with self-confidence and self-efficacy as underlying constructs using the summed score (between zero and 36) [1]. | x |  |  |  |
| Social support | - | Four questions from the subscale ‘social support in problem situations’ from the short version of the Social Support List Interaction version (SSL12-I). A summed score (between four and sixteen) was given to provide a rough indication of the amount of self-reported, received social support [2]. | x |  |  | x |
| Health care use | - | Medical and paramedical health care use in the month prior to infection, including received home care | x |  |  |  |
|  | - | Influenza vaccination prior to infection | x |  |  |  |
|  | - | Medical and paramedical health care use, including received home care, during and after the COVID-19 infection related to COVID-19 related symptoms | x |  |  |  |
|  | - | Medical and paramedical health care use, including received home care, in the past four weeks related to COVID-19 related symptoms |  | x | x | x |
|  | - | Did the received health care meet your expectations? | x | x | x | x |
|  | - | Selfcare measures taken for the treatment of COVID-19 symptoms, or lasting COVID-19 symptoms | x | x | x | x |
|  | - | COVID-19 vaccination | x | x | x | x |
| COVID-19 acute  phase | - | Certainty of COVID-19 infection | x |  |  |  |
|  | - | Approximate time of COVID-19 infection: <one month ago, one-to-three months ago, four-to-six months ago, >six months ago | x |  |  |  |
|  | - | First action upon discovering COVID-19 infection | x |  |  |  |
|  | - | Perceived severity of the COVID-19 infection: ‘I had none or hardly any symptoms’, ‘I had symptoms similar to a severe cold’, ‘I had many symptoms, but did not have to go to the hospital’ and ‘I had so many symptoms that I was hospitalized’ | x |  |  |  |
|  | - | Symptoms during the acute phase of the infection and their perceived severity (none, mild, moderate, marked, severe, or I do not know). The acute phase was defined as the initial phase following the time of infection. Participants further noted whether the symptoms were still present at the time of the questionnaire, and if yes, with which severity and how the course of the symptoms proceeded. | x |  |  |  |
| Post-COVID-Syndrome | - | The currently present symptoms, their severity (none, mild, moderate, marked, severe, or I do not know), and whether these symptoms had been present continuously since the acute phase of the infection. |  | x | x | x |
|  | - | Do you consider yourself recovered from COVID-19 |  | x | x | x |
|  | - | Do you think you have or have had the post-COVID-syndrome? |  |  |  | x |
| Quality of life | - | The Short Form Health Survey (SF-12): The SF-12 consists of 12 questions from which a physical and a mental composite score (PCS and MCS, respectively) was calculated ranging from zero-to-one-hundred, with lower scores meaning worse quality of physical or mental health [3]. In Q1 the SF-12 was also retrospectively asked for the month before the infection. | x | x | x | x |
|  | - | Perceived shortness of breath: Perceived influence of shortness of breath in daily life in the past month and in the month before the participant got COVID-19 were asked for, as well as the perceived hindrance of the infection or its effects during activities of daily living at that moment. The question on perceived shortness of breath is a core outcome measure in trials for people with COVID-19 [4]. | x | x | x | x |
|  | - | General assessment of health (now and before the COVID-19 infection) | x | x | x | x |
| Lifestyle | - | Height (cm) | x |  |  |  |
|  | - | Weight (kg) | x | x | x | x |
|  | - | Smoking status | x | x | x | x |
|  | - | Smoking history | x |  |  |  |
|  | - | Number of hours per day spent seated, and whether this was more or less than before the COVID-19 infection | x | x | x | x |
|  | - | Number of glasses of alcohol consumed | x | x | x | x |
|  | - | How often they participated in activities of moderate to vigorous intensity, and whether this was more or less than before the COVID-19 infection | x | x | x | x |
| Employment | - | Type of work at the moment of filling the questionnaire | x | x | x | x |
|  | - | Hours of payed work | x | x | x | x |
|  | - | Type of employment | x | x | x | x |
|  | - | Effects of the infection on employment: whether the participant worked more or less in the past month due to the (persistent symptoms after they got) COVID-19, whether the working activities had changed due to physical or psychological complaints, and whether they had been absent of volunteer work due to COVID-19 | x | x | x | x |

| S1B Table. Description of electronic health record data | |
| --- | --- |
| Topic | Description |
| Patient characteristics | Age |
|  | Sex |
| Medical history | ICPC-I coded (chronic) comorbidities as registered by the general practitioner and as defined by Nielen et al [5] |
| Health care use | ICPC-I coded contacts with the general practitioner and the mental health care nurse |
|  | ATC-coded prescriptions |
|  | Referrals to secondary care |
| Clinical outcomes | Measurements as recorded in the lab dossier, these include smoking status, blood pressure, lab measurements, etc. |

**References**

1. van der Meer, C.A.I., et al., *Assessing Psychological Resilience: Development and Psychometric Properties of the English and Dutch Version of the Resilience Evaluation Scale (RES).* Front Psychiatry, 2018. **9**: p. 169.

2. van Eijk, L.M., G.I. Kempen, and F.L. van Sonderen, *[A short scale for measuring social support in the elderly: the SSL12-I].* Tijdschr Gerontol Geriatr, 1994. **25**(5): p. 192-6.

3. Ware, J.E., M. Kosinski, and S.D. Keller, *SF-12: How to score the SF-12 physical and mental health summary scales.* Second edition ed. 1995: Boston, MA: The Health Institute, New England Medical Center.

4. Tong, A., et al., *Core Outcome Measures for Trials in People With Coronavirus Disease 2019: Respiratory Failure, Multiorgan Failure, Shortness of Breath, and Recovery.* Crit Care Med, 2021. **49**(3): p. 503-516.

5. Nielen, M., et al., *Berekening morbiditeitscijfers op basis van NIVEL Zorgregistraties eerste lijn*. 2016, NIVEL: Utrecht.
