# Supplementary material for: Nivel Corona Cohort: A description of the cohort and methodology used for combining general practice electronic records with patient reported outcomes to study impact of a COVID-19 infection
Source: PLoS One. 2023 Aug 22;18(8):e0288715. doi: 10.1371/journal.pone.0288715 (PMC10443834; doi:10.1371/journal.pone.0288715)
Supplement: S2 Table — (DOCX) [file pone.0288715.s003.docx]

| **S2 Table. Duration of the symptoms during the initial acute COVID-19 infection** | | | |
| --- | --- | --- | --- |
| **The symptom initially lasted longer than a week** | **N** | **% of full cohort (n=442)** | **% of those with the symptom** |
| Fatigue | 344 | 77.8 | 86.2 |
| Reduced condition | 337 | 76.2 | 86.6 |
| Anosmia / ageusia | 191 | 43.2 | 73.5 |
| Shortness of breath | 227 | 51.4 | 81.7 |
| Chest pain | 99 | 22.4 | 63.5 |
| Coughing / sneezing / stuffy nose | 221 | 50.0 | 63.5 |
| Fever | 109 | 24.7 | 40.2 |
| Nauseous / dizzy | 103 | 23.3 | 51.0 |
| Sore throat | 78 | 17.7 | 32.9 |
| Headache | 148 | 33.5 | 45.7 |
| Myalgia | 154 | 34.8 | 54.0 |
| Coughing phlegm | 113 | 25.6 | 64.2 |
| Coughing blood | 4 | 0.9 | 50.0 |
| Hyperhidrosis | 106 | 24.0 | 50.2 |
| Cold shivers | 73 | 16.5 | 30.8 |
| Vomiting | 7 | 1.6 | 18.4 |
| Stomach ache | 32 | 7.2 | 43.2 |
| Reduced appetite | 126 | 28.5 | 47.6 |
| Insomnia | 116 | 26.2 | 69.1 |
| Joint pain | 118 | 26.7 | 63.1 |
| Diarrhea | 45 | 10.2 | 46.4 |
| Trouble concentrating | 207 | 46.8 | 75.3 |
| Memory impairments / forgetfulness | 148 | 33.5 | 88.1 |
| Trouble thinking | 148 | 33.5 | 76.3 |
| Anxiety | 62 | 14.0 | 51.7 |
| Depression | 49 | 11.1 | 71.0 |
| Post-traumatic stress syndrome | 9 | 2.0 | 81.8 |
