# Supplementary material for: Nivel Corona Cohort: A description of the cohort and methodology used for combining general practice electronic records with patient reported outcomes to study impact of a COVID-19 infection
Source: PLoS One. 2023 Aug 22;18(8):e0288715. doi: 10.1371/journal.pone.0288715 (PMC10443834; doi:10.1371/journal.pone.0288715)
Supplement: Attachment — Submitted filename: Response to Reviewers.docx [file pone.0288715.s005.docx]

**Response letter	-	Revision 2 PlosONE**

*Nivel Corona Cohort: a description of the cohort and methodology used for combining general practice electronic health records with patient reported outcomes to study impact of COVID-19*

**General**

We would like to thank the reviewer and editor for the comments that helped improving the manuscript and the opportunity to resubmit the manuscript. Please find our answers to the remarks below.

**Additional Editor Comments**
Compared to the previous (first) version of the manuscript, the authors have addressed most of the remarks. A supplementary review revealed that still some elements need to be clarified. Authors are asked to address the issues raised in this review.

**Reviewer #2**

**General**

This study needs very extensive revision. The first aim of this manuscript is to describe the recruitment methodology, but the way it was presented was not satisfactory as the description was very qualitative while a clear step by step process with numbers attached would have been more transparent. The second aim was to describe the cohort and do some additional analysis. This part needs to be more focused as plenty of indicators were analyzed, but there is no clear rational for the inclusion of all this wide array of outcomes. Generally, in a cohort description, one can limit the focus to a limited number of carefully selected outcomes.

**Specific comments**
1. Title: Needs to be more specific as to the impact of COVID-19 on what ? health ? which dimension of health? Also, impact of the pandemic ? or a COVID-19 infection ?

**Response 1.: We updated our title to clarify that we study the impact of a COVID-19 infection. As we measure many different variables (work, health, quality of life) we do not further specify the title.**

2. Abstract: Suggest to add subsections for better clarity.

**Response 2.: We added subsections for clarity reasons.**

3. In the Introduction, there is an important focus on post-acute COVID-19 syndrome (PCS). However, this issue is not examined further in the paper. So, this provides some confusion as to the aim of the paper.

**Response 3.: The cohort was set up with the aim to investigate both the short- and long-term impact of a COVID-19 infection. In the introduction there was indeed a main focus on PCS. We agree with the reviewer that this is not in line with the aim of the paper and therefore shortened the paragraph on PCS.**

4. Introduction: In the abstract, the second aim was : “to examine the symptoms and healthcare usage during the acute COVID-19 phase”. However, the second aim in the introduction is much more wide and does not mention the acute phase anymore : “The second aim was to describe the characteristics of the population-based cohort of COVID19 patients, their symptoms, and healthcare usage for COVID-19 and to examine whether demographical and clinical characteristics differed between patients who perceived the severity of their infection differently.” It is necessary therefore that the aim of the paper is clarified and is consistent between the different sections of the paper.

**Response 4.: The aim of the paper is 1. to provide a description of the set-up of the cohort, including representativeness and 2. to describe the primary characteristics of the cohort on symptoms and health care usage in the acute phase. We updated the aim of the introduction to match the aim of the abstract.**

5. Method: The recruitment process needs to be described in a more transparent manner. Figure 1 is clear but the authors need to attach the numbers to it. The process has started with how many people in order to arrive at the end of the process with 442 participants ?

**Response 5.: The first paragraph in the results section describes how we arrived at 442 participants. We chose to not include numbers in figure 1 to avoid adding results to the methods section. We restructured the paragraphs so that all information on participation is now included in one paragraph.**

6. Method: The description of the questionnaire is quite lengthy and messy, more info can go to a table and it would be clearer and more interesting to the readers to have a more condensed and synthetic information about the questionnaire and the electronic record, rather than so much details in the text.

**Response 6.: We included two short paragraphs on patient reported outcomes and electronic health record data and included an additional supplementary table for the extensive description.**

7. Method: The authors need to add a rational for including and analyzing this series of variables.

**Response 7.: We included these variables to be able to perform an indepth study of the short- and long-term effects of a COVID-19 infection. Therefore we included information on determinants (such as life style, but also resilience) and on outcomes, such as quality of life, and shortness of breath in questionnaires sent to the cohort**

8. Data analysis: Representativeness is not only assessed compared to the population you have invited but to the target population, in this case for instance people who have the R83.03 code in the EHR.

**Response 8.: The target population (flagged patients minus those that participate in the Nivel corona cohort) was now also compared to the population in the Nivel corona cohort in table 1. As only 252 of the 2103 flagged patients were excluded by the GPs, the characteristics of this population were very much like those of the invited population.**

9. Data analysis: The subsection “analyses” was not clear to me.

**Response 9.: The data analysis paragraph was condensed into one paragraph including a description of both cohort representativeness and the cohort characteristics within the acute phase of the infection.**

10. Results: In Q1, the cohort includes participants with acute COVID-19 (with or without symptoms), people with long COVID, and people who have recovered. When presenting the results for instance of quality of life, health care, social support, physical activity or resilience, do they refer to the current situation or the situation during the acute phase of the disease? If the acute phase, then how was it defined ? If the current situation, then the results would be mixing these groups with very different situations.

**Response 10.: For traits that are relatively stable we asked for the current status. For several other questions (such as health care use, health status, shortness of breath, quality of life) we made a distinction between before COVID-19 and at the moment of filling in the questionnaire. This is included in supplementary table 1A. In the results section we use data from Q1 (acute phase) and included in the headings and/or subtitles of tables where applicable.**

11. Results: Table 2 has a messy format, please rearrange- Please spell out SSL12 and RES.

**Response 11.: Table 2 was rearranged and the abbreviations were spelled out.**

12. Results: Again, as mentioned above, too many outcomes and no rationale for their inclusion. For instance, why social support, hours seated, alcohol consumption, etc.. a rational for including the different outcomes analyzed in the context of this manuscript is necessary.

**Response 12.: For this paper we aimed to characterize the cohort, we did not mean to be exhaustive. Therefore, in agreement with the comment of the reviewer, we made a selection of the presented results that are in line with our research questions.**

13. Results: What is the rationale to have so detailed info on health care in Table 3?

**Response 13.: This was now removed from the paper to be concise and more in line with the research questions.**

14. Results: Severity: the authors starts the subsection with " During the acute COVID-19 phase". How is this defined ?

**Response 14.: This was based on self-report. In the questionnaire we asked for symptoms during the acute phase. This is now clarified in the methods section.**

15. Results: Prevalence of symptoms: The proportions seems very high to me, so these are during the acute phase and only for people who declared symptoms ? Because in the Severity section, 20.4% of the people indicated to have had none or hardly any symptom. So, how do we have 90% with fatigue, 88% with reduced condition, etc…

**Response 15.: Both questions were based on self-report. 90% reported to have had fatigue, however this consisted of mild, moderate, marked and severe fatigue. The latter was now included in the text.**

16. Results: Aftercare: what is meant by aftercare ?

**Response 16.: We meant to describe care after the acute phase of the COVID-19 infection. However, this was now removed from the results section as it was not required to answer the research questions.**

17. Results: Generally, there are too many subtitles, the subsections could be more integrated. For instance we do not need 3 subtitles in the data analysis section. Also, the information needs to be presented in a more integrated way as now it is very compartmentalized. For instance, why do we have a section on COVID-19 infection, severity and another about prevalence of symptoms and duration.

**Response 17.: The different sections on data analysis were now combined as were the different sections on the COVID-19 infection.**

18. Discussion: It was mainly a summary rather than a discussion

**Response 18.: The part where we summarized the results was condensed, to make it more a discussion, rather than a summary.**

19. Discussion: No discussion or mention of first aim of the study.

**Response 19.: The first aim of the study was now included in the first paragraph of the discussion.**
